# Characteristics of Soil Arsenic Contamination and the Potential of Pioneer Plants for Arsenic Remediation in Gold Mine Tailings

**DOI:** 10.3390/toxics11121025

**Published:** 2023-12-16

**Authors:** Lei Han, Yunmeng Zhai, Rui Chen, Yamin Fan, Zhao Liu, Yonghua Zhao, Risheng Li, Longfei Xia

**Affiliations:** 1 School of Land Engineering, Chang’an University, Xi’an 710054, China; 2021135017@chd.edu.cn (Y.Z.); 2021135016@chd.edu.cn (Y.F.); lz13@chd.edu.cn (Z.L.); yonghuaz@chd.edu.cn (Y.Z.); 2 School of Earth Science and Resources, Chang’an University, Xi’an 710054, China; ruichen@chd.edu.cn; 3 Shaanxi Provincial Land Engineering Construction Group, Xi’an 710075, China; lirisheng1209@163.com (R.L.); summer321xia@foxmail.com (L.X.)

**Keywords:** soil As, ecological restoration, enrichment characteristics, bioavailability, potential ecological risks, Qinling Mountains

## Abstract

Arsenic (As) contamination of gold mine tailings poses major threats to the natural environment and human health, necessitating adequate management measures. To investigate the soil As contamination level and the potential of pioneer plants for As remediation, the soil and plants of an abandoned gold mine tailings in the Qinling Mountains were analyzed. The level of As contamination was assessed using the single-factor pollution index and potential ecological risk index, and its bioeffectiveness was analyzed. The enrichment capability of plants was investigated using the bioaccumulation factor and translocation factor. Redundancy analysis and partial least squares regression were employed to investigate factors affecting the distribution of As in soil and plants. The results show that As in soil mainly existed in the difficult-available state, with serious contamination and extremely high ecological risk. *Lythrum salicaria* L. and *Equisetum ramosissimum* Desf. are the preferred plants for remediation of As contamination through screening pioneer plants. Soil total nitrogen (STN) and available phosphorus (SAP) are the main factors influencing the characteristics of As distribution in the soil. Soil available potassium (SAK), water content (SWC), and SAP promote the accumulation of As by plants. This study provides plant materials and new ideas for mine ecological remediation.

## 1. Introduction

Arsenic (As) is a highly biotoxic and carcinogenic metalloid element of great public concern [1,2]. As is designated as one of the five most hazardous elements due to its detrimental impacts on human health [3], plant and animal growth [4], and the ecological environment [5]. Environmental As is mainly released through natural processes such as the weathering of minerals and rocks and geothermal activity as well as human activities including mineral extraction and fertilizer application [6]. In general, human activities, in particular mining, are the primary cause of the large increase in As levels in the environment. Mining activities account for 72.6% of all human-caused As emissions into the environment [7]. The issue of As contamination in the soil resulting from mining activities is progressively gaining prominence [8,9]. 

As has been discovered in several gold deposits, which is a globally important precious metal mineral resource [10,11]. Resource exploitation and precious metal mining expose As hidden in mineral rocks to the environment. Gold mine tailings contain significant quantities of mining waste rock and beneficiation waste from the mining and smelting processes [12]. These wastes, through weathering, rainfall, and evaporation, can contaminate the atmosphere, soil, and water, causing various environmental issues [13,14]. There have been reports of contaminated gold mine tailings in several countries, including Portugal, China, Canada, and the UK [15,16]. Tailings are frequently regarded as priority treatment locations in the global process of ecological rehabilitation of mines [17]. Therefore, in the case of severe As contamination in gold mine tailings, it is essential to identify effective remediation strategies to address the situation.

Phytoremediation as an in situ remediation method has been demonstrated in tailing-contaminated soil [18,19]. The method takes green plants as the restoration material and uses the absorption and enrichment characteristics of plants to remove or fix heavy metals in the soil [20]. It not only beautifies the landscape but also improves the quality of the soil environment [21]. Excess As can be toxic to plant tissues and organs, impede plant growth, and even cause plant death. Therefore, the difficulty in the phytoremediation of As-contaminated soils is the discovery of tolerant and enrichment plants. Many As hyperaccumulator plants have been identified, such as *Pteris cretica* [22], *Peteris umbrosa* [23], and *Peteris multifida* [24]. The tailing soil generally exhibits high pH, a lack of nutrients, and poor soil structure, all of which lead to poor regional habitat conditions. Although hyperaccumulators can take up large amounts of As and accumulate it in their bodies, they usually have limited adaptability to environmental conditions [25]. By contrast, native plants have adapted to local soil and climate characteristics. These plants will often survive in extreme conditions more easily than introduced hyperaccumulators and will not be invasive to the local area [26,27]. Therefore, screening dominant native plants for gold mine tailings phytoremediation is a good option.

The chemical form of soil As mainly determines its environmental risk compared to its total amount [28]. As can be combined with hydrous oxides, sulfides, organic active groups, and other substances in soil, showing many speciations with different hazards. Studying the distribution traits and bioavailability of As is essential to comprehend the real extent of As contamination [29,30]. In addition, soil physicochemical properties such as pH and nutrients greatly influence the solubility and mobility of As [31,32]. They also have an impact on the process of As enrichment and transport in plants [33]. 

The Qinling Mountains is one of the world’s quintessential representative areas of biodiversity and is known as the “world’s biological gene pool” [34]. It simultaneously serves multiple functions, including environmental purification, water containment, and climate regulation [35,36]. The Qinling Mountains contain a large number of gold deposits. The destructive impacts of long-term resource exploitation and the emergence of outstanding problems left over from history have caused some damage to the ecological environment and made the task of ecological restoration very arduous. The ecological protection of the Qinling Mountains as a national strategy has become a research hotspot [37,38]. In addition to fostering the region’s sustainable development, the preservation of the ecology of the Qinling Mountains is essential to maintaining the global ecological environment. 

Previous studies on phytoremediation of As contamination have mainly focused on analyzing the total amount of soil As and plant enrichment characteristics in gold mining areas. However, understanding the physicochemical properties of regional soils and the chemical form characteristics of As is a prerequisite for improving the quality of the soil environment. Meanwhile, determining the influencing factors affecting the distribution of As in soils and plants can lead to targeted measures to improve the remediation potential of plants. Based on the above, we selected abandoned gold tailings within the Qinling Mountains in China as the research object. The main objectives of the research are (1) to analyze the soil physicochemical properties, determine the actual content of As, and evaluate the extent of soil As contamination and its potential risk; (2) to analyze the soil As fraction and effectiveness through sequential extraction; (3) to compare the difference in As content and As enrichment and transport ability in pioneer plants and use them as a basis for screening plant materials suitable for extracting and stabilizing As-contaminated soil; (4) to study the influence of physicochemical properties on the characteristics of soil As and to explore the factors influencing plant enrichment of As concentration.

## 2. Materials and Methods

### 2.1. Study Area

**Figure 1:**
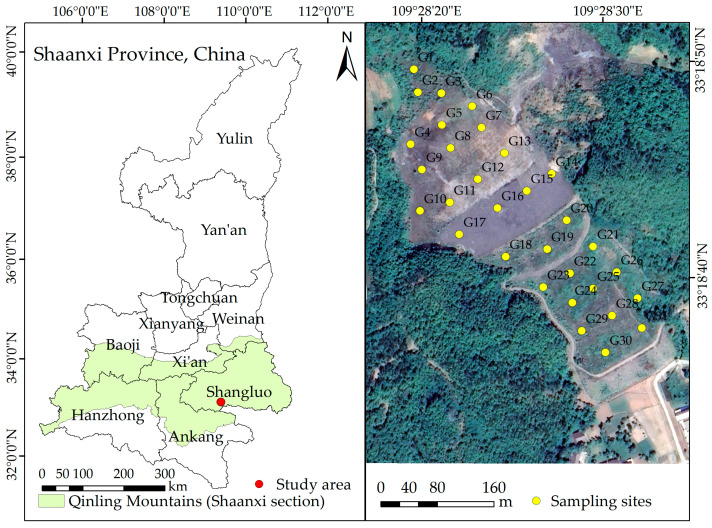
Geographical location of the study area and distribution of sampling sites.

The gold mine tailings pond is located in the Qinba Mountain area at the southern foot of the Qinling Mountains at the southwest of Shangluo City, Shaanxi Province, China (Figure 1). It is located in the South Qinling Indo-Branch Fold Belt of the Qinling Stratigraphic Fold System. The regional climate is subtropical monsoon, with a mean annual temperature of 12.2 °C, and a mean annual rainfall of 804.8 mm. The tailings pond was put into operation in 1993, and it ceased to be used after a dam failure occurred in the 2006 expansion. Due to the long-term abandonment of the tailings, the residue left behind poses a serious threat to humans and the surrounding environment. It has been identified as one of the heavy metal control areas in China. The study area’s soil type is mainly cinnamon soil, and the regional topography is highly variable, with terrain generally high in the northwest and low in the southeast. Arsenopyrite and toxic sand are the principal gold-bearing minerals in the area, and gold has a close correlation with S and particularly with As. Annual and perennial herbs and a few dwarf shrubs are the main vegetation types in the area.

### 2.2. Soil and Plant Sample Collection

Soil and vegetation growth in the tailings was investigated in the field in August 2020. The growth of dominant vegetation in the study area is shown in Figure S1. A total of 31 vegetation survey samples of 4 m × 4 m in size were laid out (Figure 1). In each sample, 2–3 plant species with a large distribution, relatively high cover, and good growth were selected. For each plant species, three whole plants of approximately the same size were collected, totaling 198 plant samples. The pioneer plant species in the study area were summarized (Table S1). The soil samples, which were collected by removing surface debris, were composed of a tiny amount of soil attached to the pioneer plant’s roots and the surrounding 0–20 cm surface layer of the soil. Each sample weighed at least 1 kg and yielded a total of 31 soil samples. These soil samples were split into three groups, one for determining physicochemical properties, one for determining As concentration, and one for determining As speciation. RTK was used to accurately record the geographical location of the sampling sites and information about their surroundings throughout the sampling process.

### 2.3. Soil and Plant Data Determination

The pH was measured using a glass electrode in a suspension with a soil-to-water ratio of 1:2.5 [39]. Soil bulk density (SBD) and soil water content (SWC) were measured using the ring knife method [40] and the gravimetric method [41], respectively. The soil organic matter (SOM), soil total potassium (STK), soil available potassium (SAK), soil total phosphorus (STP), soil available phosphorus (SAP), soil total nitrogen (STN), and soil alkaline nitrogen (SAN) were determined following Chen [42]. The As concentration in plants was digested using a microwave digestion device and measured using an atomic fluorescence photometer [43]. The As concentration in soil was detected via ICP-MS [44]. The As oxidizable speciation in soil was collected using the sequential extraction method [45]. This method is based on the modified BCR method with the addition of the determination of the water-soluble fraction that is easily absorbed and utilized by plants. As speciations in soils were classified into five categories, including a mild acidosoluble fraction (40 mL 0.11 mol·L^−1^ CH_3_COOH), a reducible fraction (40 mL 0.50 mol·L^−1^ NH_2_OH·HCl), an oxidizable fraction (10 mL H_2_O_2_, 50 mL 1 mol·L^−1^ NH_4_OAc), a residual fraction (HCl-HNO_3_-HF-HClO_4_ mixed acid solution), and a water-soluble fraction (20mL pure water, extracted alone). The extraction rate of soil As was 86.20–103.78%, the sum of each fraction was not less than 80% of the test value, and the standard deviation of parallel samples was within 5%, which met the requirements.

### 2.4. Evaluation of Soil As Contamination and Its Biological Effectiveness

#### 2.4.1. Single-Factor Contaminant Index (Pi)

Pi is a widely used method for evaluating the contamination degree of heavy metal elements in soil [46]. Its calculation formula is as follows:

(1) $$P_{i}=C_{i}/S_{i},$$

where Pi is the single-factor contamination index of heavy metal element i; C_i_ is the measured value of heavy metal element i, mg·kg^−1^; and Si is the standard or background value of that heavy metal element in soil, mg·kg^−1^. In this study, the screening value of soil As contamination risk in the second type of development land in China (60 mg·kg^−1^) was used as the standard value [47]. The classification of Pi is shown in Table S2.

#### 2.4.2. Potential Ecological Risk Index (PERI)

The PERI was proposed by Swedish geochemist Hakanson in 1980 [48]. It is mainly used to quantitatively evaluate the potential ecological hazard level of heavy metal elements in the soil. This method takes into account the enrichment degree of heavy metal elements and unique physiological toxicity [49]. The calculation formula is as follows:

(2) $$E_{r}=T_{0}\times (C_{i}/B_{i}),$$

where Er is the potential ecological hazard index for heavy metal i; T_0_ is the toxic response coefficient of heavy metals, where As is generally considered to be 10 [50]; Ci is the measured value of heavy metal elements in the soil, mg·kg^−1^; Bi is the environmental background value of heavy metal elements in the soil, mg·kg^−1^; and the soil As in Shaanxi Province of China is 11.2 mg·kg^−1^ [51]. The grading is shown in Table S2.

#### 2.4.3. Biological Activity Factor

Heavy metals are typically present in different speciations in the soil and their activity and toxicity are highly variable. The biological activity factor can be used to evaluate the effects of heavy metals on the soil environment and organisms. They can be classified into an available state (K_1_), medium-available state (K_2_), and difficult-available state (K_3_) according to their fugitive speciations [52]. The calculation formula is as follows:

(3) $$K_{1}=\left(F_{1}+F_{5}\right)/\left(F_{1}+F_{2}+F_{3}+F_{4}+F_{5}\right),$$

(4) $$K_{2}=\left(F_{2}+F_{3}\right)/\left(F_{1}+F_{2}+F_{3}+F_{4}+F_{5}\right),$$

(5) $$K_{3}=F_{4}/\left(F_{1}+F_{2}+F_{3}+F_{4}+F_{5}\right),$$

where K_1_, K_2_, and K_3_ are biological activity coefficients; and F_1_–F_5_ are extraction speciations of heavy metals, where F_1_ is the mild acidosoluble fraction, F_2_ is the reducible fraction, F_3_ is the oxidizable fraction, F_4_ is the residual fraction, and F_5_ is the water-soluble fraction.

### 2.5. Enrichment and Transport Ability of Plants for As

#### 2.5.1. Bioaccumulation Factor (BCF)

The BCF is the ratio of heavy metal content in the above-ground part of the plant to the corresponding heavy metal content in the soil [53]. It has been used to assess plants’ ability to take up heavy metals from the soil [54]. The calculation formula is as follows:

(6) $$\mathrm{BCF}=\;B_{1}/B_{2},$$

where B_1_ is the heavy metal content in the above-ground part of the plant, mg·kg^−1^; and B_2_ is the content of that heavy metal in soil, mg·kg^−1^.

#### 2.5.2. Translocation Factor (TF)

The TF refers to the ratio of heavy metal content in the above-ground part of plants to the same heavy metal content in the below-ground part of plants [55]. It has been used to assess plants’ ability to transport heavy metals from their below-ground to their above-ground parts [56]. Its calculation formula is as follows:

(7) $$\mathrm{TF}=\;C_{1}/C_{2}$$

where C_1_ is the content of heavy metals in the above-ground part of the plant, mg·kg^−1^; and C_2_ is the content of heavy metals in the below-ground part of the plant, mg·kg^−1^.

### 2.6. Analysis of Influencing Factors

#### 2.6.1. Pearson Correlation Analysis and Redundancy Analysis (RDA)

The linear relationship between two continuous variables can be examined using Pearson correlation analysis. The correlation between physicochemical properties and soil As characteristics was calculated in this study using Pearson correlation analysis, and the physicochemical indicators that showed a significant link with soil As characteristics were identified. RDA is a gradient ranking analysis method that combines regression analysis with principal component analysis. It can statistically analyze the relationship between one set of variables and another set of variables. In this study, different As fractions and total As were used as response variables, and physicochemical indicators were used as explanatory variables to analyze the degree of contribution of each physicochemical property to soil As characteristics and to identify the key determinants.

#### 2.6.2. Partial Least Squares Regression Model (PLSR)

Considering the small number of samples and the possible multicollinearity between variables, a PLSR was applied to analyze the mechanism of soil environmental factors (SWC, SBD, SOM, SAP, SAK, SAN, and different fractions of As) affecting the As content in plants, and key environmental factors were screened out based on the analysis results. The explanatory power of the independent variables on the dependent variables was measured by the variable predictive importance index (VIP value).

### 2.7. Data Statistics and Analysis

The study area and sampling site location markers were mapped using ArcGIS 10.7 software (Esri, Redlands, CA, USA). Data pre-processing was performed using Excel 2016 software (Microsoft, Redmond, WA, USA), and graphics were made using Origin 2021 software (OriginLab, Northampton, MA, USA). Pearson correlation analysis was performed using SPSS26 software (IBM, Armonk, NY, USA). The redundancy analysis (RDA) was performed using Canoco5.0 software (Microcomputer Power, Ithaca, NY, USA), and the partial least squares regression model (PLSR) was applied in SMICA14.1 software (MKS Umetrics, Umeå, Vasterbottens Lan, Sweden).

## 3. Results

### 3.1. Soil Physicochemical Characteristics

The determination results of the physicochemical properties of the tailing soil samples are shown in Table 1. It was found that the soil pH value ranged from 7.53 to 8.87, with a mean of 8.41, and the soil was generally mildly alkaline to alkaline. The SWC varied between 4.01% and 40.81%. The mean value of SBD was 1.33 g·cm^−3^, and the soil texture was tight. The differences in spatial heterogeneity of the soil physicochemical properties were analyzed using coefficient of variation values (CV) [57]. The only variable with a CV of less than 10% was pH, which showed little variation in its spatial distribution. The CV values of the remaining variables ranged from 14.29% to 87.92%, showing moderate variability. The nutrient quality of tailing soils was evaluated according to the Chinese soil nutrient grading standard (Table S3). Among them, STP, SOM, SAN, and SAK were in deficiency, STN and SAP were moderate, and STK was in the rich category. On the whole, the tailing soil nutrients were insufficient, with wide variations in spatial distribution.

### 3.2. Characterization of Soil As Contamination

#### 3.2.1. Speciation and Concentration of As in Soil

**Figure 2:**
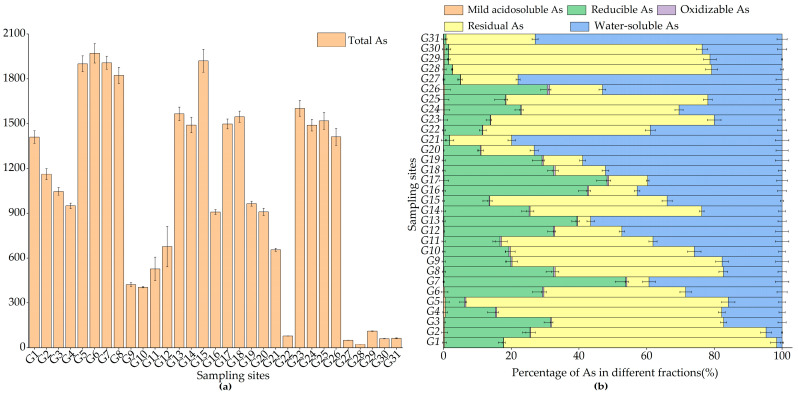
Distribution of total As (**a**) and the proportion of different speciations of As (**b**) in the studied soil.

Figure 2 and Table 2 show the distribution of total soil As and the proportion of each speciation. As seen in Figure 2a and Table 2, the variation of total soil As ranged from 22.08 to 1971.54 mg·kg^−1^. The total soil As was high and the spatial distribution varied greatly, which was 1.97–176.03 times the background value of the local soil environment. In Figure 2b and Table 2, the proportion of As varied considerably among different speciations. It mainly manifested as the residue, reducible, and water-soluble fractions, and the sum of the items belonging to these three speciations was about 98.96–99.93% of the total amount. The residual As was in the range of 3.87–80.65% with a mean value of 42.78%, which accounted for the largest proportion, followed by water-soluble As with 35.92% on the mean. The smallest proportion was made up of mild acidosoluble As, at only 0.01% to 0.52%.

#### 3.2.2. Soil As Contamination Evaluation and Bioavailability Analysis

**Figure 3:**
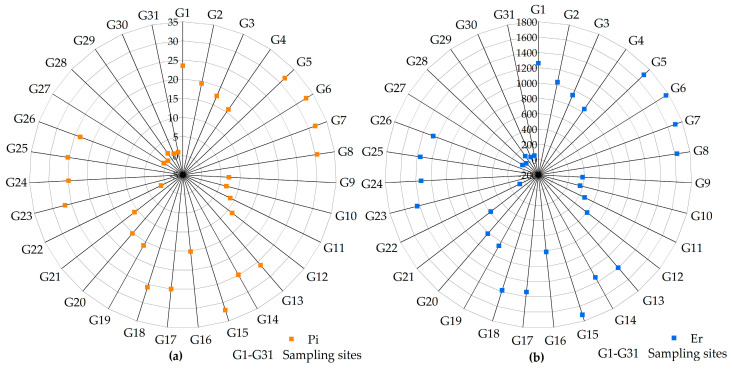
Pi (**a**) and PERI (**b**) evaluation results of As contamination in the studied soil.

Figure 3 displays the evaluation results of the As contamination levels in the surface soil of the tailings. The Pi of the soil As ranged from 0.37 to 32.86, and there were three grades of contamination: severe, sight, and no pollution, among which the degree of contamination was severe at 25 points. The PERI was calculated as ranging from 19.71 to 1760.31 for soil As, with moderate, considerable, and extremely high ecological risk, with 25 sampling points having extreme ecological risk. As detailed in Table S4, the bioactivity coefficients of soil As, K_1_, K_2_, and K_3_ were 0.36, 0.21, and 0.43, respectively, demonstrating that K_3_ > K_1_ > K_2_. As was mostly present in the difficult-available state, followed by the available state, and the least in the medium-available state. This indicates that the soil As was more stable, less susceptible to environmental changes, and difficult to be released into the soil for bioreuse.

#### 3.2.3. Correlation between Soil As and Physicochemical Properties

**Figure 4:**
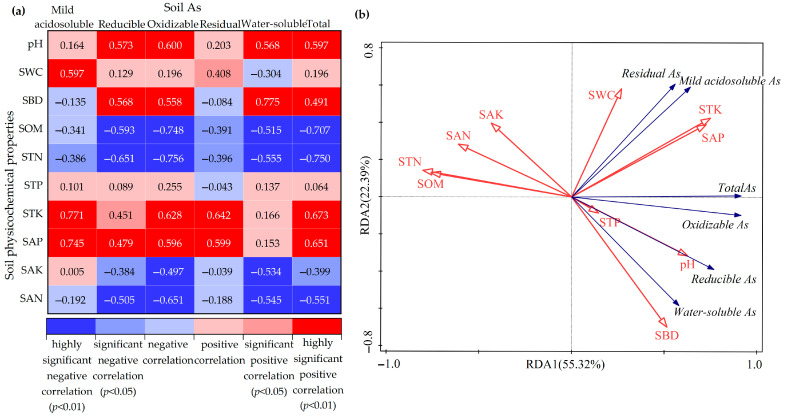
Correlation analysis (**a**) and RDA analysis (**b**) of soil As and physicochemical properties. (In Figure 4b, red arrows represent soil physicochemical properties and blue arrows represent soil As characteristics).

Figure 4 displays the outcomes of the Pearson correlation analysis and RDA between the soil’s physicochemical properties and each fraction of As and total As. The correlation between soil each fraction of As and total As and physicochemical properties was basically consistent, as seen in Figure 4a. There was a significant correlation between As and pH, SOM, STN, and other indicators, showing that these physicochemical properties had a major impact on the presence of soil As. The pattern of As presence in the soil was positively correlated with pH, STK, and SAP and negatively correlated with SOM, STN, and SAN. As seen in Figure 4b, the findings show that the influence of different soil physicochemical properties on each fraction of As and total As was variable, with STN, SAP, SBD, SAN, STK, pH, SOM, SWC, SAK, and STP in descending order. The soil’s chemical characteristics, of which STN explained 36.2% and SAP explained 15.4%, had a greater influence on soil As than its physical characteristics.

### 3.3. As Characterization of Pioneer Plants

#### 3.3.1. As Concentration in Pioneer Plants

**Figure 5:**
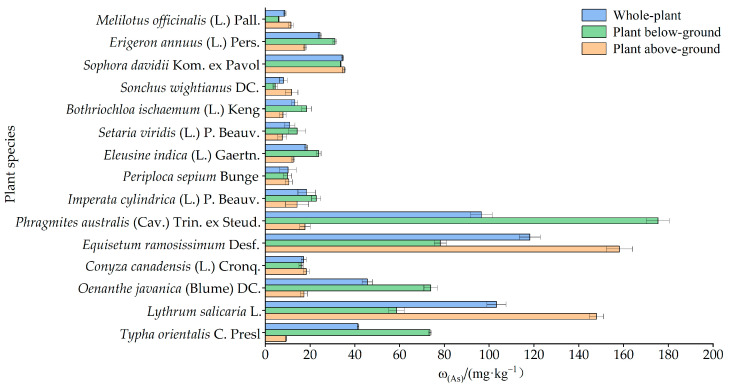
Distribution of As contents in the whole, above-ground, and below-ground parts of pioneer plants.

Figure 5 depicts the As contents in the whole, above-ground, and below-ground parts of the 15 pioneer plants. There was some variation in the enriched As among different plants and in different parts of the same plant. For the whole plant, the As content in plants ranged from 8.11 to 118.17 mg·kg^−1^, with *Equisetum ramosissimum* Desf. (Content_As_ = 118.17 mg·kg^−1^), *Lythrum salicaria* L. (Content_As_ = 103.27 mg·kg^−1^), and *Phragmites australis* (Cav.) Trin. ex Steud. (Content_As_ = 96.58 mg·kg^−1^) being significantly higher than other plants. In terms of plant parts, the As accumulation in the above-ground parts of the 15 pioneer plants was between 7.61 and 158.14 mg·kg^−1^, with a mean value of 33.17 mg·kg^−1^ and ranged in the below-ground parts from 4.46 to 175.38 mg·kg^−1^, with a mean value of 42.68 mg·kg^−1^. Common plants typically have an As content of less than 5 mg·kg^−1^ [58]. Despite having low As contents, the 15 pioneer plants in the tailings still had higher As contents than common plants.

#### 3.3.2. Analysis of Pioneer Plants’ Ability to Enrich and Transport As

The BCFs and TFs of As by the pioneer plants in the tailings are shown in Table 3. The results show that the BCFs and TFs of various plants for As varied considerably, with values for BCFs ranging from 0.006 to 0.447 and BTFs from 0.101 to 2.637. *Lythrum salicaria* L. had a BCF value of 0.447 for As, which was the highest value of all pioneer plants and shows a good enrichment ability, followed by *Equisetum ramosissimum* Desf. and *Phragmites australis* (Cav.) Trin. ex Steud., with BCF values of 0.263 and 0.119, respectively. Except for these three plants, all other plants’ BCFs were smaller than 0.1, indicating that they had relatively poor BCFs for As. Among the pioneer plants, the TFs of *Sonchus wightianus* DC and *Lythrum salicaria* L were 2.637 and 2.523, respectively, indicating that they had a high As transport ability, while the TF of As was the worst for *Imperata cylindrica* (L.) P. Beauv, with a value of 0.101. The TFs for As of seven pioneer plants such as *Lythrum salicaria* L. were greater than 1. Their method of survival involves moving the harmful element As from the plant’s below-ground part to its above-ground part. This decreases the toxicity of As to the below-ground part and increases the competitiveness of plants in the survival process when collecting nutrients from the soil. The TFs of the remaining pioneer plants for As were smaller than 1. This indicates that these plants showed tolerant growth by preventing the transport of As from the below-ground parts to the above-ground parts of the plants through their growth exclusion mechanism.

#### 3.3.3. Correlation of As Levels in Pioneer Plants with Soil Environmental Factors

**Figure 6:**
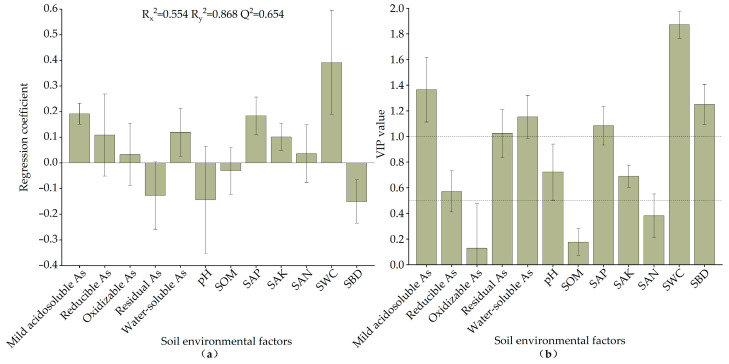
Regression coefficients and their confidence intervals (**a**) and VIP values of the independent variables and their 95% confidence intervals (**b**).

In the PLSR method analysis, both R^2^ and Q^2^ exceeded 0.5, indicating a satisfactory model fit. When the Q^2^ regression line’s intersection with the vertical axis is negative after 200 substitution tests, there is no overfitting of the model and the model is considered verified. The regression results make it possible to find the regression coefficient and the independent variable projected importance index (VIP values), which can intuitively reflect the role of soil environmental factors on the effect of plant As content. From Figure 6a, mild acidosoluble As, reducible As, oxidizable As, water-soluble As, SAP, SAK, SAN, and SWC were positively correlated with the As contents in the studied plants, and the rest of the variables were negatively correlated with them. As shown in Figure 6b, the VIP values, in descending order, were SWC, mild acidosoluble As, SBD, water-soluble As, SAP, residual As, pH, SAK, reducible As, SAN, SOM, and oxidizable As. The mild acidosoluble As, water-soluble As, residual As, SAP, SWC, and SBD had VIP values greater than 1, indicating that they were the main influences on the As content absorbed by plants. The reducible As, pH, and SAK had VIP values greater than 0.5, indicating that they were significant influences. The VIP values of the remaining variables were smaller than 0.5, indicating that they had a minor role in influencing As enrichment by plants.

## 4. Discussion

### 4.1. Effects of Physicochemical Properties on As Characterization in Soil

Environmental factors such as pH affect the content and speciation of heavy metals in soil by influencing their transferability and bioavailability [59]. In this study, pH, SBD, STK, and SAP showed significant and highly significant positive correlations with partial speciations of As and total As, whereas SOM, STN, SAK, and SAN showed significant and highly significant negative correlations. This shows that within a given range, an increase in soil pH, SBD, STK, and SAP had a beneficial effect on the partial speciations of As and total As, whereas SOM, STN, SAK, and SAN had an inhibitory effect on them. The correlation between pH, total As, and water-soluble As was significantly positive. An increase in soil pH will weaken the adsorption of As, causing both the total and the water-soluble As contents in the soil solution to rise [60]. The results of correlation analyses between SOM and As content have varied among scholars [61,62]. In this study, SOM was highly and significantly negatively correlated with As content. This is because an increase in SOM would reduce the sorption of As by the soil and promote the migration of As, resulting in a decrease in soil As content [63]. The results of the RDA revealed that STN and SAP had the greatest impact on the As fractions and the total As in soil. This indicates that N and P are important factors affecting the distribution of various As fractions and the total As in the soil. This may be because of elements’ absorption and transfer between soil and plants, as well as biogeochemical behaviors such as soil ammonification and nitrification that affect As [64,65]. In soil, phosphorus (P) and As have a symbiotic or competitive relationship, while in alkaline soil, P is better at adsorbing than As [66]. Therefore, increasing the concentration of P to a certain extent will reduce the adsorption capacity of soil for As and increase the resolved quantity of As.

### 4.2. Classification of the Accumulation Characteristics of Pioneer Plants for As

In general, 25 mg·kg^−1^ of soil As is the upper limit for normal plant growth (soil pH > 7.5) [57]. All 15 pioneer plants could grow normally even though the As levels in the tailing soil were over this threshold, demonstrating that these plants had adapted to the environment. Most of the pioneer plants had a low BCF for As but a high vegetation cover. These pioneer plants had a strong tolerance to soil As after a period of evolution and natural selection [67]. Plants can be classified into three types: accumulators, excluders, and root compartments depending on their mechanisms of heavy metals tolerance [68]. The results of the classification are shown in Table S5. *Lythrum salicaria* L. and *Equisetum ramosissimum* Desf. have high As contents and strong As transport ability, so they have the characteristics of accumulators. The As content in the below-ground parts of the two plants *Typha orientalis* C. Presl and *Oenanthe javanica* (Blume) DC. is high, and their TFs are less than 1, which has the characteristics of root compartments. The remaining 11 pioneer plants including *Sonchus wightianus* DC. have lower As content and can grow stably in the soil of the tailing soil, with the characteristics of excluders.

### 4.3. Factors Influencing As Enrichment in Plants

Soil physicochemical properties play a certain role in the process of plant enrichment of As [69]. Among the soil As characteristics, the mild acidosoluble As and water-soluble As have a high bioavailability and mobility and are the principal speciations taken up by plants [42]. Consequently, as their concentration grows, so does plant absorption of As. When it comes to physicochemical properties, SAP and SWC promote plant uptake of As to some extent; however, pH and SBD are negative. P, to some extent, promotes the amount of As resolved in the soil, and also offers substances needed for plant growth, thus enhancing the uptake of As by the plant. pH is closely related to the bioavailability of As and the accumulation of As in plants [70]. Although an increase in pH can improve the bioavailability of soil As [71], too high a pH value is detrimental to plant growth [72]. In this study, an increase in soil pH resulted in a decrease in As accumulation in plants. Relevant studies have demonstrated that variations in SWC, one of the soil’s key characteristics, affect other physicochemical characteristics (pH, Eh, etc.) [73]. High SWC is accompanied by low SBD, poor soil aeration, and low Eh values. This condition encourages the reductive decomposition of Fe/Mn oxides, which releases As into the soil environment for plant reutilization [74]. In addition, it encourages plant metabolism growth and development processes when there is enough water in the soil.

### 4.4. Measures for Remediation of As-Contaminated Tailing

The soil environment of the gold mine tailings found through the investigation is generally alkaline, with low nutrient contents and poor soil quality and structure. It must be improved before phytoremediation can be carried out. Among the pioneer plants, *Lythrum salicaria* L. and *Equisetum ramosissimum* Desf. had high As concentrations, and both had better translocation capacity. Additionally, both of them had the advantages of high vitality, strong stress resistance, and large biomass. They can be the preferred plants for soil As contamination extraction remediation. However, due to the poor bioavailability of soil As in the tailings, the bioavailability of As can be increased by using activators as well as regulating soil physicochemical properties to improve the uptake of As by plants [75,76]. Gradual removal of As from the soil is achieved by continuous planting and harvesting of plants with enrichment characteristics. Some root compartments and excluders can be selectively planted around the mining area. This will not only improve the local ecological environment but also reduce the negative impact of As entering humans and other animals through the food chain.

### 4.5. Deficiency and Outlook

Plant materials suitable for ecological remediation of As-containing gold mines were screened in this study. The results can be used as a basis for exploring planting patterns or cooperative remediation techniques in the actual restoration process to achieve the regional construction goals (preventing soil erosion and purifying the soil environment). In addition, the relationship between the speciation and total amount of As in the soil and its physicochemical properties, as well as the effect of soil environmental factors on the enrichment of As in plants, were discussed. This can be used as a basis to regulate the soil environment and improve the plant enrichment capacity in the actual remediation process. However, the mechanisms of As enrichment and transport by plants and the interaction mechanisms between soil physicochemical properties and As were not discussed in detail. Furthermore, the potential applications of As-accumulating plants can be explored, for example, whether these plants can be used as adsorbents for environmental protection and health after carbonization. These issues can be further investigated in the future.

## 5. Conclusions

As contamination from gold mining tailings has been an environmental problem of great concern worldwide. We conducted a systematic study on the soil and vegetation of abandoned gold mine tailings. The nutrient quality of the surface soil of the study area was poor, with low TP, SOM, SAN, and SAK contents. The soil As levels all exceeded the limits for normal plant growth, and its contamination levels were serious, with more than moderate ecological risk. The mild acidosoluble As and water-soluble As in the soil were the main speciations of As absorbed in plants. STN and SAP played a major role in the distribution of As in the soil, whereas SAN, STK, and pH also had some degree of influence. SWC, SBD, and SAP are the key environmental factors for the ability of plants to uptake As. Thus, the bioavailability of soil As and plants’ ability to accumulate As can be improved to some extent by adjusting the soil’s physicochemical properties. *Lythrum salicaria* L. and *Equisetum ramosissimum* Desf. can be used for the extraction remediation of tailings remediation. Root compartments and excluders are preferred species for soil and water conservation and ecological environment maintenance in and around tailings. This study screened plant species used for the remediation of soil As contamination and analyzed the effect of physicochemical properties on soil As characteristics, as well as the effect of both soil physicochemical properties and As characteristics on plant enrichment of As. The findings can serve as a theoretical foundation for the development of regional restoration work. They can also guide the remediation of soils in similar mining areas and complement the phytoremediation database.

## Figures and Tables

**Table 1:** Basic physicochemical properties of soils.

Index	Max (mg·kg^−1^)	Min (mg·kg^−1^)	Mean (mg·kg^−1^)	Standard Deviation	Coefficient of Variation (%)
pH	8.87	7.53	8.41	0.26	3.09
SWC (%)	40.81	4.01	15.02	0.11	73.33
SBD (g·cm^−3^)	1.81	1.03	1.33	0.19	14.29
SOM (g·kg^−1^)	5.18	0.03	1.49	1.31	87.92
STN (g·kg^−1^)	3.58	0.42	1.25	0.80	64.00
SAN (mg·kg^−1^)	200.60	20.04	65.12	42.51	65.28
STP(g·kg^−1^)	0.10	0.02	0.04	0.02	50.00
SAP (mg·kg-^1^)	24.97	3.05	10.77	6.47	60.07
STK (g·kg^−1^)	29.50	10.51	21.24	4.68	22.03
SAK (mg·kg^−1^)	232.09	23.44	72.88	54.41	74.66

**Table 2:** Soil As concentration and percentage of different forms of As.

As Characteristics	Min (mg·kg^−1^)	Max (mg·kg^−1^)	Mean (mg·kg^−1^)	Coefficient of Variation (%)	Percentage (%)	Mean Percentage (%)
Mild acidosoluble As	0.06	9.13	1.85	117.03	0.01–0.52	0.17
Reducible As	0.38	721.89	238.30	87.25	0.60–53.72	20.84
Oxidizable As	0.02	10.15	3.44	83.79	0.03–0.72	0.29
Residual As	8.48	1472.57	422.92	93.84	3.87–80.65	42.78
Water-soluble As	4.59	1024.77	347.90	82.70	1.58–79.85	35.92
Total	22.08	1971.54	1034.41	63.22	–	–

**Table 3:** BCFs and TFs of pioneer plants for As.

Plant Species	BCF	TF
*Typha orientalis* C. Presl	0.037	0.126
*Lythrum salicaria* L.	0.447	2.523
*Oenanthe javanica* (Blume) DC.	0.042	0.233
*Equisetum ramosissimum* Desf.	0.263	1.145
*Phragmites australis* (Cav.) Trin. ex Steud.	0.119	2.203
*Imperata cylindrica* (L.) P. Beauv.	0.035	0.101
*Eleusine indica* (L.) Gaertn.	0.034	0.624
*Setaria viridis* (L.) P. Beauv.	0.015	1.068
*Bothriochloa ischaemum* (L.) Keng	0.009	0.522
*Sophora davidii* Kom. ex Pavol	0.047	0.533
*Melilotus officinalis* (L.) Pall.	0.023	0.428
*Sonchus wightianus* DC.	0.009	2.637
*Conyza canadensis* (L.) Cronq.	0.009	1.048
*Erigeron annuus* (L.) Pers.	0.016	0.571
*Periploca sepium* Bunge	0.006	1.918

## Data Availability

Data are contained within the article and supplementary materials.

## Supplementary Materials

The following supporting information can be downloaded at https://www.mdpi.com/article/10.3390/toxics11121025/s1: Figure S1: Growth of pioneer vegetation in the study area; Table S1: Pioneer plant species in the study area. Table S2: The classification of Pi and PERI; Table S3: Soil nutrient classifications in China; Table S4: Soil As bioactivity activity coefficient; Table S5: Classification of pioneer plants.
