# Supplementary material for: Characteristics of Soil Arsenic Contamination and the Potential of Pioneer Plants for Arsenic Remediation in Gold Mine Tailings
Source: Toxics. 2023 Dec 16;11(12):1025. doi: 10.3390/toxics11121025 (PMC10747858; doi:10.3390/toxics11121025)
Supplement: Supplementary file 1 [file toxics-11-01025-s001.zip › toxics-2759698-supplementary.pdf]

toxics

MDPI

Supplementary Materials: Characteristics of Soil Arsenic Contamination and the Potential of Pioneer Plants for Arsenic Remediation in Gold Mine Tailings

Lei Han, Yunmeng Zhai, Rui Chen, Yamin Fan, Zhao Liu, Yonghua Zhao, Risheng Li and Longfei Xia

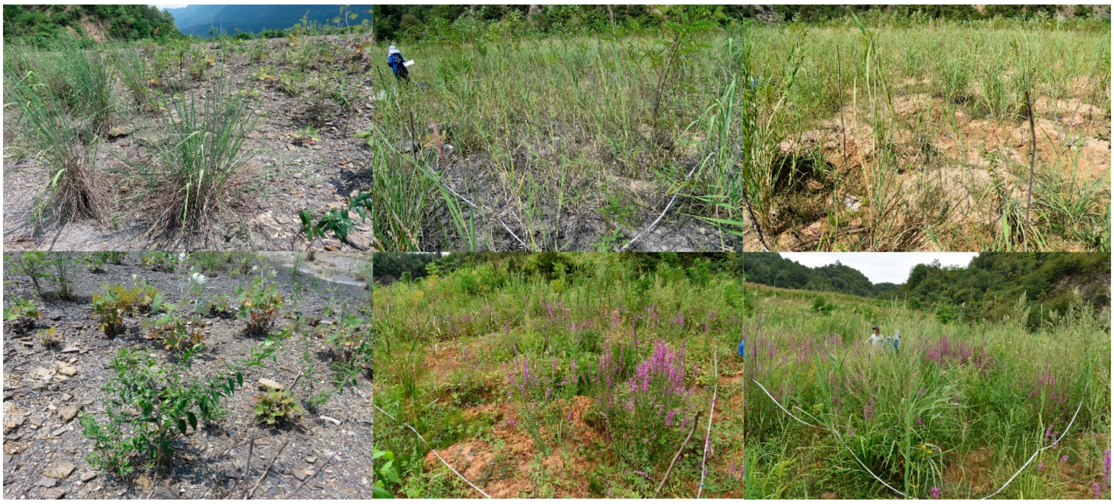

Figure S1. Growth of dominant vegetation in the study area.

Table S1. Pioneer plant species in the study area.

Plant name	Genus	Family	Lifeform
Typha orientalis C. Presl	Typha Linn.	Typhaceae	Herb
Lythrum salicaria L.	Lythrum L.	Lythraceae	Herb
Oenanthe javanica (Blume) DC.	Oenanthe L.	Apiaceae	Herb
Equisetum ramosissimum Desf.	Equisetum L.	Equisetaceae	Herb
Phragmites australis (Cav.) Trin. ex Steud.	Phragmites	Poaceae	Herb
Imperata cylindrica (L.) P. Beauv.	Imperata Cyr.	Poaceae	Herb
Eleusine indica (L.) Gaertn.	Eleusine Gaertn.	Poaceae	Herb
Setaria viridis (L.) P. Beauv.	Beauv. Ess. Agrost.	Poaceae	Herb
Bothriochloa ischaemum (L.) Keng	Bothriochloa Kuntze	Poaceae	Herb
Sophora davidii Kom. ex Pavol	Styphnolobium Schott	Leguminosae	Shrub
Melilotus officinalis (L.) Pall.	Melilotus Mill.	Leguminosae	Herb
Sonchus wightianus DC.	Sonchus	Asteraceae	Herb
Conyza canadensis (L.) Cronq.	ConyzaLess.	Asteraceae	Herb
Erigeron annuus (L.) Pers.	Erigeron L.	Asteraceae	Herb
Periploca sepium Bunge	Periploca	Asclepiadaceae	Shrub

Table S2. The classification of Pi and PERI.

Pi	Pollution level	Er	Degree of ecological risk
Pi ≤ 1	No pollution	Ei＜40	Low ecological risk
1＜Pi ≤ 2	Sight pollution	40 ≤ Ei＜80	Moderate ecological risk
2＜Pi ≤ 3	Light pollution	80 ≤ Ei＜160	Considerable ecological risk
3＜Pi ≤ 5	Middle pollution	160 ≤ Ei＜320	Very high ecological risk
Pi ≥ 5	Severe pollution	Ei ≥ 320	Extremely high ecological risk

Table S3. Soil nutrient classifications in China.

Level	1<br>Very rich	2<br>Rich	3<br>Moderate	4<br>Lack of	5<br>Very lacking	6<br>Extremely lacking
SOM($g·kg^{-1}$)	>40.00	30.00-40.00	20.00-30.00	10.00-20.00	6.00-10.00	<6.00
STN ($g·kg^{-1}$)	>2.00	1.50-2.00	1.00-1.50	0.75-1.00	0.50-0.75	<0.50
SAN($mg·kg^{-1}$)	>150.00	120.00-150.00	90.00-120.00	60.00-90.00	30.00-60.00	<30.00
STP($g·kg^{-1}$)	>1.00	0.80-1.00	0.60-0.80	0.40-0.60	0.20-0.40	<0.20
SAP($mg·kg^{-1}$)	>40.00	20.00-40.00	10.00-20.00	5.00-10.00	3.00-5.00	<3.00
STK($g·kg^{-1}$)	>20.00	15.00-20.00	10.00-15.00	5.00-10.00	3.00-5.00	<3.00
SAK($mg·kg^{-1}$)	>200	150.00-200.00	100.00-150.00	50.00-100.00	30.00-50.00	<30.00

Table S4. Soil As bioactivity activity coefficient.

Biological activity coefficient	K_1	K_2	K_3
As	0.36	0.21	0.43

Table S5. Classification of pioneer plants.

Classification	Plant species
Accumulators	*Lythrum salicaria* L.
	Equisetum ramosissimum Desf.
Root compartments	*Typha orientalis* C. Presl
	Oenanthe javanica (Blume) DC.
Excluders	*Phragmites australis* (Cav.) Trin. ex Steud.
	Imperata cylindrica (L.) P. Beauv.
	Eleusine indica (L.) Gaertn.
	Setaria viridis (L.) P. Beauv.
	Bothriochloa ischaemum (L.) Keng
	Sophora davidii Kom. ex Pavol
	Melilotus officinalis (L.) Pall.
	Sonchus wightianus DC.
	Conyza canadensis (L.) Cronq.
	Erigeron annuus (L.) Pers.
	Periploca sepium Bunge
